# Murine model for congenital CMV infection and hearing impairment

**DOI:** 10.1186/1743-422X-8-70

**Published:** 2011-02-15

**Authors:** Chen Juanjuan, Feng Yan, Chen Li, Liu Haizhi, Wang Ling, Wang Xinrong, Xiao Juan, Liu Tao, Yin Zongzhi, Chen Suhua

**Affiliations:** 1 Department of Obstetrics and Gynecology, Affiliated Tongji Hospital, Tongji Medical College, Huazhong University of Science and Technology, Wuhan, PR China; 2 Department of Obstetrics and Gynecology, The First Affiliated Hospital of Fujian Medical University, Fujian, PR China; 3 Department of Obstetrics and Gynecology, The First Affiliated Hospital of Xiamen University, Xiamen, PR China; 4 Department of Obstetrics and Gynecology, The first affiliated Hospital of Jinan University, Guangzhou, PR China; 5 Department of Obstetrics and Gynecology, Affiliated Yuhuangding Hospital, Qingdao University, Qingdao, PR China

## Abstract

**Background:**

Congenital cytomegalovirus (CMV) infection is the leading cause of sensorineural hearing loss (SNHL), and SNHL is the most frequent sequela of congenital CMV infection. But the pathogenic mechanism remains unknown, and there is no ideal CMV intrauterine infection animal model to study the mechanisms by which SNHL develops.

**Methods:**

We established the congenital murine cytomegalovirus (MCMV) infection model by directly injecting the virus into the placenta on day 12.5 of gestation. Then, we observed the development and the MCMV congenital infection rate of the fetuses on the day they were born. Furthermore, we detected the auditory functions, the conditions of the MCMV infection, and the histological change of the inner ears of 28-day-old and 70-day-old offspring.

**Results:**

Both the fetal loss rate and the teratism rate of offspring whose placentas were inoculated with MCMV increased, and their body length, head circumference, and weight decreased. The hearing level of offspring both decreased at both 28- and 70-days post birth; the 70-day-old mice developed lower hearing levels than did the 28-day old mice. No significant inflammatory changes in the cochleae of the mice were observed. MCMV DNA signals were mainly detected in the spiral ganglion neurons and the endolymph area, but not in the perilymph area. The number of neurons decreased, and their ultrastructures changed. Moreover, with age, the number of neurons dramatically decreased, and the ultrastructural lesions of neurons became much more severe.

**Conclusions:**

The results suggest that the direct injection of MCMV into the placenta may efficiently cause fetal infection and disturb the intrauterine development of the fetus, and placental inoculation itself has no obvious adverse effects on offspring. The reduction in the number of spiral ganglion neurons and the ultrastructural lesions of the neurons may be the major cause of congenital CMV infection-induced progressive SNHL.

## Background

Human cytomegalovirus (HCMV) is the most common pathogen causing intrauterine infection in developing countries [1]. Pregnant women are susceptible to primary and secondary HCMV infections. If a pregnant woman is infected with HCMV, the virus can be transmitted through the placenta or cervix, causing intrauterine infection.

In developed countries, current estimates indicate that [2,3] about 0.64% to 0.7% of all children are born with HCMV. The incidence of HCMV is higher (1.2%) in societies with low socioeconomic status, regardless of whether pregnant women are infected. Only 11% to 12.7% of children with congenital HCMV infection exhibit clinical signs at birth, such as petechia, hyperbilirubinemia, hepatosplenomegaly, thrombocytopenia, chorioretinitis, seizures, microencephaly, intracranial calcification, fetal edema, etc. However, approximately 40% to 58% of children with symptomatic infection and 13.5% of children with asymptomatic infection develop long-term neurological sequelae, including single- or two-sided progressive sensorineural hearing loss (SNHL), cognitive deficit, and motor deficit. In a clinical epidemiological study of 5,015 pregnant women and their children from big urban centers in China [4], we found that 5.42% of the pregnant women were actively infected with HCMV and 37.32% of the newborns from actively infected pregnant women were congenitally infected with the virus. The infected children seem to be at higher risk for hearing loss, mental retardation, and other developmental anomalies of the nervous system during infancy and childhood [5]. Li et al. [6] found that the intellectual development, especially the language skills development, of early childhood and preschool children with asymptomatic intrauterine HCMV infection is evidently lower than that of normal children. In addition, the pure-tone threshold of preschool children with asymptomatic intrauterine HCMV infection is abnormal.

To date [7], it is believed that SNHL is the most frequent sequela of congenital CMV infection and that congenital CMV infection is the leading cause of SNHL. Approximately 15%-25% of congenital hearing loss cases in children are caused by intrauterine HCMV infection [8-10] and 10%-20% of children with congenital CMV infection exhibit varying degrees of hearing loss [11-13]. Theoretically, SNHL caused by intrauterine HCMV infection may be closely related to inner ear diseases, but the exact pathogenic mechanism remains unknown.

The pathogenic mechanism remains unknown primarily because of the lack of an ideal animal model for thorough studies. Similar to humans, the placenta of guinea pigs has a single trophoblast layer that separates maternal and fetal circulations. Hence, the guinea pig cytomegalovirus (GPCMV) can cross the guinea pig placenta, causing infection in utero [14,15]. However, the relatively lengthy gestational periods (65-70 d) and the lower-than-average litter size (2-4 newborn pups) of guinea pigs [16] result in longer and, consequently, costly experimental cycles. More importantly, the lack of available guinea pig immunological reagents critically hampers the development of research on congenital GPCMV infection and hearing loss.

In studying how intrauterine HCMV infection causes SNHL, various models, including transgenic and knockout animals, are available. Mice have significantly shorter gestational periods(19-21 d) and significantly greater litter size (10-12 newborn pups). The murine cytomegalovirus (MCMV) genome is highly similar to the HCMV genome, and the characteristics of CMV infection in mice are comparable to human CMV infection. Therefore, the congenital MCMV infection model is highly useful in the development of research on intrauterine CMV infection-induced hearing loss.

Two studies [17,18], utilizing the congenital GPCMV infection model, have demonstrated the following: (a) congenital GPCMV infection results in hearing loss and severe labyrinthitis in newborns; and (b) GPCMV-infected cells are mainly located in the perilymph area and spiral ganglion, not in the endolymph area. However, the long-term pathology of congenitally infected offspring, including ultrastructural changes remains unknown. Moreover, no study has been conducted on congenital CMV infection-induced hearing loss using the murine model. The placental barrier is presumed to be refractory to CMV transmission in mice because murine placenta has a three-cell-thick trophoblast layer that separates maternal and fetal circulations; hence, we established a congenital murine cytomegalovirus infection model by directly injecting MCMV to the placenta, as performed by Dr. Y Tsutsui [19]. We used the model to observe the growth of offspring before and after birth, especially the long-term auditory functions, the conditions of the MCMV infection, and the long-term histological changes of their inner ears. The purpose of this study is to establish an ideal animal model of CMV intrauterine infection, and use this model to preliminarily study the possible mechanisms of MCMV congenital infection-induced hearing loss.

## Methods

### Virus and cell cultures

The MCMV Smith strain was purchased from the American Type Culture Collection (ATCC, VR-1399). The strain was passaged in mouse embryonic fibroblasts (MEF). MEF cells were prepared from 12-day-old embryos of BALB/C mice and grown in Dulbecco's modified Eagle's minimal essential medium (DMEM), which contained penicillin (100 U/ml), streptomycin (50 ug/ml), and 10% fetal calf serum (FCS). The plaque assay method of Wentworth and French was used to quantify the virus. The titer of the virus stock was 1 × 10^6 ^plaque-forming units (PFU)/ml. The virus samples were stored in aliquots (-70°C), and each aliquot was frozen and thawed only once.

### Intraplacental infection of mouse embryos with MCMV

Specific-pathogen-free (SPF) BALB/C mice [12 ± 1 w, 22 ± 2 g (female), 26 ± 2 g (male)] were obtained from Tongji Medical College of Huazhong University of Science and Technology (Hubei, China). The mice without MCMV infection [IgG(-) and IgM(-)], as identified by enzyme-linked immunosorbent assay, served as subjects. The male and female mice were placed in the same cage at a male-female ratio of 1:2. The onset of pregnancies, when copulation plugs were found after overnight mating, was marked as day 0. Plugged females were housed individually. On day 12.5 of gestation, the pregnant mice were operated on under anesthesia with 10% chloral hydrate (350 mg/kg).

The pregnant mice were randomly divided into 3 groups: the experimental group (n = 22), in which the number of gestational sacs were counted, and then 1 ul of the 1 × 10^6 ^per ml stock was injected into the placentas using a microsyringe; the operation control group (n = 24), in which the number of gestational sacs were counted, and then 1 ul of 3% FCS DMEM was injected into the placentas in the same way; and the blank control group (n = 24), in which the number of gestational sacs was counted and no other actions were performed.

After the procedure, the fetuses of some pregnant mice [experimental group (n = 12), operation control group (n = 12), and blank control group (n = 12)] were allowed to develop in uteru. They were surgically collected on day 18.5 of gestation. We then observed the conditions and the development of the intrauterine infection. First, we counted the number of fetuses, including the number of live fetuses and dead fetuses; observed the abnormalities of the fetuses; measured the body length, the head circumference, and the body weight of the fetuses; and calculated the live birth rate, the fetal loss rate, and the microcephalus rate. Second, to detect the infection of the placentas and fetuses, we amplified the MCMV DNA using the polymerase chain reaction (PCR). The placentas, brains, lungs, livers, and kidneys that were sterilely collected from the fetuses were frozen at -70°C for DNA amplification using PCR.

The fetuses of other pregnant mice [experimental group (n = 10), operation control group (n = 12), and blank control group (n = 12)] were naturally delivered. We then observed the auditory functions, the conditions of the MCMV infection, and the histological changes of the inner ears of 28-day-old and 70-day-old offspring.

All animal procedures were approved by the International Council on Laboratory Animal Science and were conducted according to the Regulations for the Administration of Affairs Concerning Experimental Animals.

### Amplification of viral DNA

With regard to the detection of the viral genome in infected mice fetuses and placentas, specific DNA sequences were amplified using PCR, as described by Chen Li et al. [20]. Briefly, a set of oligonucleotide primers from exon 4 of the MCMV immediate-early (IE) gene (GenBank accession number M11788) was designed based on published sequence data [21]^. ^The 30-bp primer, which amplified a 700-bp segment of MCMV, was used. The base sequences were as follows: forward primer, 5'-ATC AAT CAG CCA TCA ACT CTG CTA CCA CAC-3' (exon 4 of the MCMV IE cDNA 1701-1730); and reverse primer, 5'-ATG GTG AAG CTA TCA AAG ATG TGC ATC TCA-3' (exon 4 of the MCMV IE cDNA 2400-2371). Template DNA was prepared through a series of phenol-chloroform extractions, and 1 ug of sample DNA was added to the reaction mixture (25 ul) before amplification. The final concentrations of components in 25 ul of the PCR reaction mixture were as follows: forward primer (0.5 ul), reverse primer (0.5 ul), template DNA (1 ul), 2 × Masterin (12.5 ul) [10 mM Tris-HCl (pH = 8.3), 50 mM KCl, 1.5 mM MgCl_2_, 250 mM d NTP, and 1 ul Taq DNA polymerase], and ddH_2_O (10.5 ul). PCR was performed as follows: Step 1--10 min at 94°C × 1 cycle; Step 2--1.5 min at 94°C, 1 min at 55°C, 2 min at 72°C × 30 cycles; and Step 3--10 min at 72°C × 1 cycle and soaked at 4°C. The products underwent electrophoresis on 1.5% agarose gels, stained with ethidium bromide, and photographed. Randomly selected PCR positive products were sent to Shanghai Sangon Company (Shanghai Sangon Biological Engineering Technology & Service Corporation Ltd., Shanghai, China; lot number SK1322) for DNA sequencing.

### Auditory brainstem response

Auditory brainstem response (ABR) was assessed to examine the effects of intrauterine MCMV infection in the inner ears of infected mice. The fetuses of the three groups were delivered naturally. The ABR of all offspring [experimental group (n = 33), randomly selected fetuses of the operation control group (n = 40), and the control group (n = 40)] were detected. Some of the offspring [experimental group (n = 16), operation control group (n = 20), and blank control group (n = 20)] were examined 28 d after delivery; the others [experimental group (n = 17), operation control group (n = 20), and blank control group (n = 20)] were examined 70 d after delivery. Needle electrodes were placed subcutaneously under tribromoethanol anesthesia (300 mg/kg, ip). For each mouse, active electrodes were inserted beneath the skin on top of the head, and the reference electrode was inserted beneath the pinna of the measured ear and the ground beneath the skin of the upper part of the nose. The test used a rapid onset, transient click stimulus rate of 11.1 clicks/second, lasting for 0.1 ms. Filter settings were at 150-2000 Hz, with a gain of 1000. The scan cycle was 10 ms. At the beginning of the test, the click intensity was reduced to 10 dB sound pressure level (SPL). The click intensity was reduced to 5 dB SPL steps as it approached the threshold. The threshold was defined as the lowest intensity level at which a clear ABR waveform was visible in the evoked trace. This was determined by visual inspection of the responses.

### Pathological cochlear examination

After the ABR tests, the cochleae were separately taken out under tribromoethanol anesthesia. Prior to the light microscope analysis, the cochleae were perfused with 4% paraformaldehyde and fixed for 24 h. After fixation, the tissues were decalcified with 10% tetrasodium EDTA in PBS (pH = 7.4) for 1 wk. The tissues were then dehydrated with gradient alcohol (75%, 80%, 90%, 95%, and 100%), cleared with butyl alcohol, embedded in paraffin, sectioned horizontally along the modiolus, and stained with hematoxylin and eosin (HE). Pathological changes were observed, and neurons in the spiral ganglia of the cochleae were counted under a light microscope.

Prior to the electron microscope analysis, the cochleae were fixed with 2.5% glutaraldehyde phosphate for 6 h at 4°C. After fixation, the tissues were decalcified with 10% tetrasodium EDTA in PBS (pH = 7.4) for 1 wk. The tissues were then fixed with 1% osmium tetroxide for 15 min, dehydrated with gradient alcohol (50%, 70%, 80%, 90%, 95%, and 100%) and acetone (90% and 100%), embedded in Epon 812, polymerized in the aggregator for 24 h at 37°C and for 24 h at 60°C, sectioned horizontally along the modiolus, and stained with 2% uranyl acetate and lead citrate. The observed ultrastructural changes were photographed.

### In situ hybridization

A synthetic oligonucleotide probe (Shanghai Sangon Biological Engineering Technology & Services Co., Ltd., China) with the following sequences were used: 5'-CCA-GAC-TCT-CTT-TTC-TGA-GGG-CCC-TAG-ATT-3' (exon 4 of the MCMV IE cDNA 1871-1900, GenBank accession number M11788). It was labeled by 5'-tailing with DIG-11-Dutp. The sections were dewaxed in water with xylene and gradient alcohol (100%, 95%, 90%, 80%, and 75%). Thereafter, the sections were treated with 3% H_2_O_2 _for 10 min and protease K (20 ug/ml) for 20 min at 37°C. Nonradioactive ISH was performed using the Enhanced Sensitive ISH Detection Kit (POD) (MK1030, Boster Biological Technology, Ltd., China) as specified by the manufacturer. The ISH signal was colored with DAB and observed through a microscope.

Quantification of the staining intensity of MCMV IE DNA was performed through image analysis. Gray scale images of MCMV IE DNA staining were recorded using an Olympus BH-2 digital camera. The HPIAS-1000 image program was used to analyze the images, and the staining intensity of the MCMV IE DNA was calculated as an optical density (OD) value.

## Results

### 1. Infection of mouse fetuses by intraplacental MCMV injection

**Figure 1:**
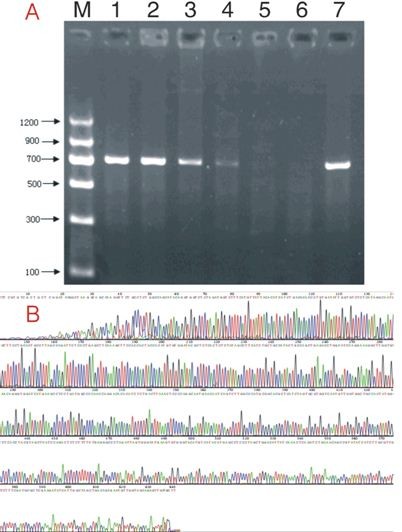
**PCR detection of MCMV genomic DNA in fetal organs and placentas from pregnant mice whose placentas were injected with MCMV, 3% FCS DMEM, or without injection**. Primers from exon 4 of the MCMV IE gene (GenBank accession number M11788) were used, yielding a 700-bp product. A: Lanes 1-4, DNA from the fetal organs or the placentas of pregnant mice whose placentas were injected with MCMV; Lane 5, DNA from the fetal organs or the placentas of pregnant mice whose placentas were injected with 3% FCS DMEM; Lane 6, DNA from the fetal organs or the placentas of pregnant mice without placental injection; Lane 7, DNA from virus-containing culture fluid. B: DNA sequence of PCR products from organs or placentas with placental MCMV injection.

To obtain an experimental animal model of intrauterine MCMV infection-induced hearing loss, we performed direct placental MCMV infection in mice fetuses at day 12.5 of gestation, a period of active neurogenesis. On day 18.5, we sacrificed part of the pregnant mice, and observed the development and the congenital infection rate of the fetuses. At day 18.5 of gestation, viral DNA amplification using PCR (the electrophoretogram of the MCMV DNA is shown in Figure 1) in the placenta was observed at 58.49% (31/53). The infection rates of the brains, livers, lungs, and kidneys of the fetuses from placentas injected with MCMV were 37.74% (20/53), 32.08% (17/53), 22.64% (12/53), and 22.64% (12/53), respectively. Fetuses infected in these organs accounted for 37.74% (20/53) of the fetuses with placental injection; the rate of fetal infection from the infected placentas was 61.29% (19/31). These results suggest that the direct injection of MCMV into the placenta may efficiently cause fetal infection.

The developmental conditions of the fetuses can be seen in Tables 1 and 2. The fetal loss rate (34.57%) and the microcephalus rate (33.96%) of the experimental group were both higher than those of the operation control group (1.21% and 2.41%, respectively) and the blank control group (1.27% and 0%, respectively). The body length and head circumference of the fetuses in the experimental group were both shorter than those of the fetuses in the other two groups, and the weight of the fetuses in the experimental group was also less than that of the fetuses in the other two groups. However, there was no significant difference in the parameters between the operation control group and the blank control group. All these data suggest that placental injection itself is not related to fetal loss, microcephalus, and the abnormal intrauterine physical growth of the fetuses, but the direct injection of MCMV into the placenta may increase the risks.

**Table 1:** Growth and developmental status of offspring

	Gestational sac (12.5 d)	Live fetuses (18.5 d)		Fetal loss (18.5 d) (absorption, abortion, and stillbirth)		Microcephalus (18.5 d)	
		
	No.	No.	Percentage (%)	No.	Percentage (%)	No.	Percentage (%)
Experimental group	81	53	65.43^1, 3^	28	34.57^1, 3^	18	33.96^1, 3^
Operation control group	83	82	98.79^2^	1	1.21^2^	2	2.41^2^
Blank control group	79	78	98.73	1	1.27	0	0

^1^p < 0.05, experimental group versus operation control group

^2^p > 0.05, operation control group versus blank control group

^3^p < 0.05, experimental group versus blank control group

**Table 2:** Physical growth status of live offspring

	No. of live fetuses	**Body length (**$\bar{x}$**± s mm)**	**Head circumference (**$\bar{x}$**± s mm)**	**Weight (**$\bar{x}$**± s g)**
Experimental group	53	25.28 ± 1.77^1, 3^	22.09 ± 2.35^1,3^	1.26 ± 0.11^1, 3^
Operation control group	82	26.29 ± 1.70^2^	23.98 ± 1.99^2^	1.41 ± 0.13^2^
Blank control group	78	26.27 ± 1.82	24.37 ± 1.74	1.41 ± 0.12

^1^p < 0.01, experimental group versus operation control group

^2^p > 0.05, operation control group versus blank control group

^3^p < 0.01, experimental group versus blank control group

### 2. MCMV-induced hearing loss in infected mice

After placental injection, some of the fetuses were allowed to develop in uterus, and they were naturally delivered. We used ABR to investigate the baseline hearing threshold of each offspring 28 d and 70 d after delivery. Thereafter, the offspring were sacrificed. The mean SPL of the offspring can be seen in Table 3. At 28 days from delivery, the mean SPL of the experimental group was significantly higher than that of the operation control group and the blank control group. Similarly, at 70 days after delivery, the mean SPL of the experimental group was significantly higher than that of the operation control group and the blank control group. However, at 28 days and 70 days after delivery, there were no significant differences between mice in the operation control group and the blank control group. Furthermore, in all three groups, there were no significant differences between 28-day-old and 70-day-old mice. The results indicate that placental injection does not lead to hearing loss in offspring; however, the direct injection of MCMV into the placenta may cause hearing loss in offspring. The injury lasts for 70 days or even longer.

**Table 3:** The mean SPL of the offspring

	28-days-old		70-days-old	
	
	No.	Mean SPL	No.	Mean SPL
Experimental group	16	36.56 ± 9.44^1,3,4^	17	40.29 ± 9.6^1, 3^
Operation control group	20	19.75 ± 3.8^2,4^	20	22.25 ± 4.13^2^
Blank control group	20	19.25 ± 3.73^4^	20	21.25 ± 3.93

^1^p < 0.01, experimental group versus operation control group

^2^p > 0.05, operation control group versus blank control group

^3^p < 0.01, experimental group versus blank control group

^4^p > 0.05, 28-days-old versus 70-days-old

### 3. Spread of virus in the cochleae of offspring in the experimental group

**Figure 2:**
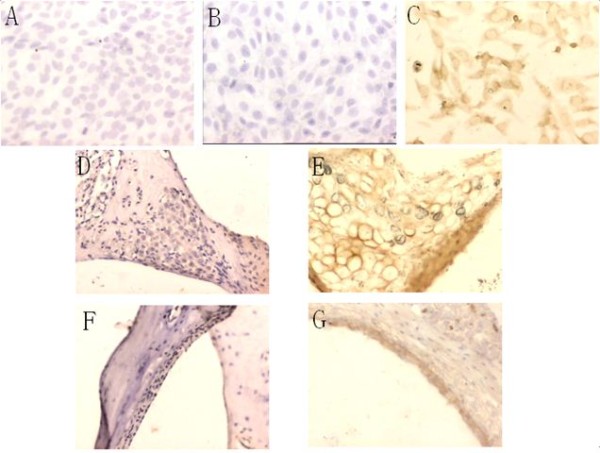
**In situ hybridization of MCMV DNA in the cochleae of congenital MCMV-infected mice**. The ISH signals are both negative in cultured cells without probe and MCMV infection (A) and cultured cells without MCMV infection (B); MCMV DNA signals are detected in the cytoplasm and the nucleus of MCMV-infected cultured cells as brown granules(C); MCMV DNA signals are negative in the spiral ganglion neurons (D) and the stria vascularis (F) of the control mice; MCMV DNA signals are detected in the spiral ganglion neurons (E) and the stria vascularis (G) of the congenital MCMV-infected mice.

After the ABR tests, the cochleae were taken out separately and processed into paraffin sections. We detected MCMV DNA signals through ISH (Figure 2). Negative ISH signals were observed in cultured cells without probe and MCMV infection, and cultured cells without MCMV infection; MCMV DNA signals were detected in the cytoplasm and the nucleus of MCMV-infected cell-climbing films as brown granules. This suggests the perfect specificity of the experiment. ISH revealed that mice in the 28-day-old offspring group and the 70-day-old offspring group had similar localizations of MCMV DNA in the cochleae. MCMV DNA signals were detected in spiral ganglion neurons and in the endolymph area, such as in the stria vascularis and spiral ligament, but not in the perilymph area. In all instances, when the MCMV DNA signal was detected in the stria vascularis and spiral ligament, it was also detected in the spiral ganglion. MCMV DNA signals were detected in 50% (16/32) of the cochleae of the 28-day-old mice [50% (16/32) in the spiral ganglion neurons, 31.25% (10/32) in the stria vascularis, and 25% (8/32) in the spiral ligaments]. For the 70-day-old mice, the positive rate of the cochleae was 38.24% (13/34), and the positive rates of the spiral ganglion neurons, stria vascularis, and the spiral ligaments were 38.24% (13/34), 17.65% (6/34), and 23.53% (8/34), respectively.

The OD values of MCMV DNA signal in the spiral ganglion neurons, stria vascularis, and spiral ligaments of the 28-day-old mice were 0.2529 ± 0.1281, 0.3112 ± 0.0980, and 0.2770 ± 0.1148, respectively. For the 70-day-old mice, the OD values of the areas were 0.4052 ± 0.2205, 0.3954 ± 0.2389, and 0.3258 ± 0.2063, respectively. There were no significant differences among the three sites in both age groups. The OD value of the spiral ganglion neurons of the 70-day-old group was significantly higher than that of the 28-day-old group. Although the OD values of the stria vascularis and spiral ligaments of the 70-day-old group were higher than those of the 28-day-old group, there were, nevertheless, no significant differences.

All these data suggest that MCMV are mainly located in the spiral ganglion neurons, then in the stria vascularis and spiral ligaments of cochleae, respectively. There were no significant differences in viral DNA content among the three sites in both 28-day-old and 70-day-old mice, although viral DNA content, especially in the spiral ganglia, increased with age (from 28 days to 70 days).

### 4. Histological changes of the cochleae of MCMV-infected mice

**Figure 3:**
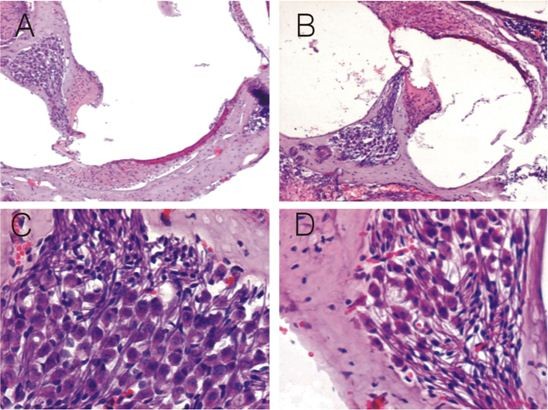
**Histology of the cochleae of normal fetuses and fetuses with congenital MCMV infection under HE staining**. At low magnification (×100), there are no obvious inflammatory lesions in the cochleae of congenitally infected fetuses (B), but in contrast to normal fetuses (A), the number of spiral ganglion neurons is decreased, and the space among neurons is widened. At high magnification (×400), in contrast to normal fetuses (C), the number of spiral ganglion neurons in the cochleae of congenitally infected fetuses is decreased (D), and the space among them is widened.

According to the morphological results detected by light microscope (Figure 3), spaces among spiral ganglion neurons were widened, and the number of neurons was reduced in both 28-day-old and 70-day-old groups. However, there were no obvious inflammatory lesions. In the 28-day-old group, there were 55.75 ± 2.357 and 60.50 ± 2.726 neuron cells in the MCMV DNA positive and negative cochleae, respectively (P < 0.01). In the 70-day-old group, there were 53.17 ± 1.169 and 57.50 ± 2.991 neuron cells in the MCMV DNA positive and negative cochleae, respectively (P < 0.01). There were fewer neuron cells in the MCMV DNA positive and negative cochleae of 70-day-old mice than those of the 28-day-old mice (P < 0.050), especially in the MCMV DNA positive cochleae.

**Figure 4:**
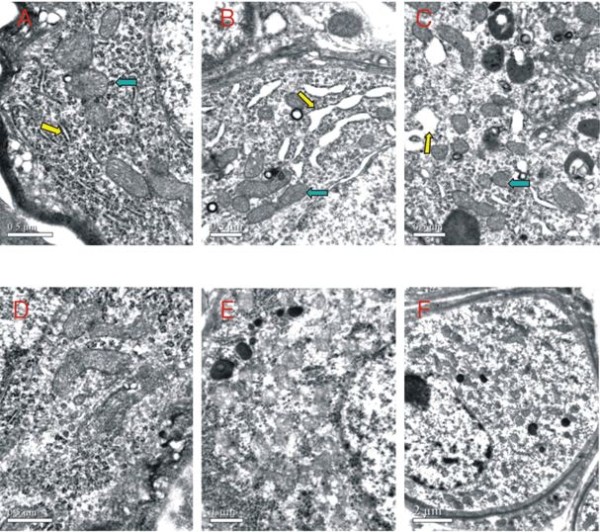
**Ultrastructure of the cochlear spiral ganglion neurons of normal fetuses and fetuses with congenital MCMV infection at different ages under electron microscope**. Compared with the normal 28-day-old pups (A), swollen endoplasmic reticula (yellow arrow) and increased lysosomes (blue arrow) can be seen in the spiral ganglion neurons of congenitally infected pups (B, C). Compared with the normal 70-day-old pups (D), the spiral ganglion neurons of congenitally infected pups (E, F) show diffused swelling, the physical component of the cytoplasm has disappeared, and a large number of floc and vacuoles has appeared. The number of lysosomes has increased. The chromatins appear condensed and hyperchromatic.

Under an electron microscope (Figure 4), the primary pathological changes in the 28-day-old mice were located in the neurons of their spiral ganglia. The endoplasmic reticula and the ribosomes were bubbly swollen, and their numbers were reduced. The number of lysosomes increased. No changes in the mitochondria were observed. Gaps in the nuclear membranes increased.

Pathological changes in the 70-day-old mice were also located in the neurons of their spiral ganglia. We found that pathological changes in the 70-day-old mice were more severe than in the 28-day-old mice. The neurons showed diffused swelling, the physical component of cytoplasm was significantly reduced, and a large number of floc and vacuoles appeared. The residual endoplasmic reticula and ribosomes were bubbly swollen. The number of lysosomes increased. Mitochondria were swollen in different degrees, and mitochondrial cristae were disordered and fractured. Chromatin was condensed and hyperchromatic.

The histological changes of the intrauterine MCMV-infected cochleae of offspring suggest that the lesions are mainly in the spiral ganglion neurons; with age, the neural lesions progress and increase, and the number of neurons decreases while exhibiting ongoing damage. This is consistent with the trend of the cochlear viral DNA content.

## Discussion

HCMV is the most common pathogen causing congenital infection worldwide. Although congenital HCMV infection can cause encephalopathy, along with cognitive, motor, auditory, or visual impairments, SNHL remains the most frequent sequela of congenital HCMV infection, and it is usually the only sequela in asymptomatic children [7]. Congenital HCMV infection-induced SNHL affects 22% to 65% of symptomatic infants and 6% to 23% of asymptomatic infants [22-24]. Epidemiological evidence also suggests that congenital HCMV infection is the leading cause of SNHL, and it is responsible for approximately 15% to 25% of congenital deafness in children [8-10]. HCMV-induced hearing loss may be present at birth, or it may occur later in the first years of life; the onset of hearing loss in over 55% of children who have hearing loss is delayed after the newborn period. The median age of the onset of hearing loss for symptomatic and asymptomatic children with delayed hearing loss is 33 months (range, 6-197 months) and 44 months (range, 24-182 months), respectively. Approximately, 50% of all children with HCMV-related SNHL will experience progression or further deterioration of their hearing loss over time [8]. We also found that congenital intrauterine HCMV infection remarkably increases the incidence of brainstem auditry in 4-month-old children. In asymptomatic infants, brainstem auditry may be under-diagnosed at the early postnatal stage because SNHL is progressive and it has a late onset [5]. Hence, we cannot provide early intervention for affected children. No effective medical or surgical therapy can cure SNHL. Cochlear implants can restore partial hearing; however, the ideal subjects of cochlear implantation are those suffering from post-linguistic deafness and the costs are so high, so the method is not widely used in China. Hence, the linguistic and communication competence of patients with CMV or congenital infection-induced SHNL cannot develop well. Therefore, it is very important to conduct an intensive study on the pathogenesis of congenital HCMV infection-induced SNHL.

In one study, the pathology of the inner ears was obtained from an infant who died of severe congenital CMV infection [25]. There was severe labyrinthitis in the vestibular endolymphatic system, especially in the saccule and utricle, with minor involvement of the cochlea, which was mainly manifested as hydrops at the basal turn. Inclusion-bearing cells that contain the CMV antigen were present on the endolymphatic surface of the membranous walls, mainly in the saccule and utricle. CMV was also isolated from the perilymph.

However, CMV was not isolated from the inner ear fluid of a 14-year-old male who died of sequela of congenital cytomegalic inclusion disease [26]. An examination of the temporal bones revealed the chronic pathology of both cochlear and vestibular tissues, with endolymphatic hydrops in the basal turn of the cochlear duct, whereas Reissner's membrane was collapsed in the more apical turns. In addition, strial atrophy and loss of cochlear hair cells along the entire length of the basilar membrane were observed.

A PCR study proved that HCMV DNA can be detected in the perilymph of patients with SNHL [27]. To better understand the damage caused by CMV infection of the inner ear, as well as its consequence and pathogenesis, experimental animal models should be used. A study on labyrinthitis following congenital transmission, which resulted from the injection of GPCMV into 5-week-pregnant guinea pigs, has demonstrated that congenital GPCMV infection results in severe labyrinthitis in fetuses. GPCMV-infected cells were detected in the perilymph area and spiral ganglion, but not in the endolymph area and the hair cells [17]. Now, by autopsy and animal models, it has been validated that congenital HCMV infection-induced SNHL is closely related to lesions in the inner ear, but the specific mechanism remains unclear.

The guinea pig model is the best animal model to be used in the study of congenital CMV infection, and congenital infection can lead to labyrinthitis in fetuses. However, the lack of available guinea pig immunological reagents suggests that animal models are not very suitable in investigating the mechanism of hearing loss. Nevertheless, mouse, the most widely used experimental animal, has a lot of available reagents. Although the three-cell-thick trophoblast layer of the murine placenta restrains the spread of MCMV to the fetus, the research of Dr. Y Tsutsui. [19] showed that the direct injection of MCMV into the placenta can cause congenital MCMV infection.

We performed direct placental MCMV infection in mice fetuses at day 12.5 of gestation, a period of active neurogenesis. The mice were sacrificed six days later. We chose to inject the virus into the placentas at day 12.5 for several reasons: (a) early blastocyst and ES cells are refractory to MCMV infection until day 7.5 [28]; (b) it is much easier to perform abortion if we make the injection before day 12.5 because the placenta is so small; (c) day 12.5 is a period of active neurogenesis. We observed the conditions and the development of the intrauterine infection. We found that 58.49% of the placentas and 37.73% of the fetuses were infected; the rate of fetal infection from the infected placentas was 58.06%. As observed, among the organs infected by MCMV, the brain tissue was the organ most sensitive to MCMV, with the highest incidence (37.73%) of infections, followed by the liver (32.07%), lung (22.64%), and kidney (22.64%). Essentially, our observations in this study are consistent with previously reported results [19]. We also observed the developmental conditions of the fetuses. Both the fetal loss rate and the microcephalus rate of the offspring from placentas inoculated with MCMV increased, and the body length, head circumference, and weight of the fetuses decreased. However, the inoculation of placentas with the same dose of 3% FCS DMEM had no significant adverse effect on offspring. These results indicate that the direct injection of MCMV into the placenta may efficiently cause fetal infection and disturb the intrauterine development of fetuses, but the inoculation itself has no obvious adverse effects on offspring. Therefore, the application of placental MCMV inoculation method has been successfully established through the mouse model.

We applied the murine model of intrauterine MCMV infection to study cochlear infection, the postnatal histological changes caused by the infection, and the auditory functions of offspring. ISH demonstrated that MCMV DNA signals can be detected in the cochleae of 28-day- and 70-day-old mice, and are mainly located in spiral ganglion neurons, and then in the stria vascularis and spiral ligaments, respectively, but not in the perilymph area. We did not observe significant inflammatory changes in the cochleae of mice across different ages. This is inconsistent with the findings of Katano et al [18]. Such inconsistency is probably due to differences in experimental methods and in the age of offspring used in the experiments. With increasing age, the inflammatory response of cochleae disappears. We further observed the pathological changes of the infected spiral ganglion neurons and found that the infection significantly decreases the number of neurons and significantly changes the ultrastructures of neurons (e.g., there is swelling of the endoplasmic reticula, ribosomes, and mitochondria). Moreover, with increasing age, the number of neurons remarkably decreases and ultrastructural lesions in some neurons become much more severe. This is consistent with the finding that the hearing level of offspring in the experimental group decreased and worsened with age. However, across different ages, the hearing level of offspring in the operation control group was equal to that of offspring in the blank control group. All the data demonstrate that the direct injection of MCMV to the placenta may cause MCMV infection of the cochleae of offspring, which leads to hearing impairment. However, the injury is not related to placental injection itself. It may be closely related to the infection of the spiral ganglion neurons, to the reduction in the number of neurons, and to ultrastructural lesions of the neurons. Pathological changes of the spiral ganglion neurons will inevitably affect their protein synthesis and secretion functions, leading to the interruption of the auditory pathway, which may be the major cause of SNHL through congenital infection. In the future, we will further study the mechanisms and possible treatment of congenital CMV infection-induced hearing loss using the same animal model.

## Competing interests

The authors declare that they have no competing interests.

## Authors' contributions

CSH, CJJ, FY and WXR participated in the design of the study. CJJ, FY, CL, LHZ and WL performed the experiment. CSH, CJJ, XJ, LT and YZZ participated in drafting the manuscript. All authors read and approved the final draft.

## References

[B1] Franca CM Mugayar LR Intrauterine infections: a literature review Spec Care Dentist 2004 24 5 250 3 10.1111/j.1754-4505.2004.tb01701.x 15552342

[B2] Dollard SC Grosse SD Ross DS New estimates of the prevalence of neurological and sensory sequelae and mortality associated with congenital cytomegalovirus infection Rev Med Virol 2007 17 5 355 363 10.1002/rmv.544 17542052

[B3] Kenneson A Cannon MJ Review and meta-analysis of the epidemiology of congenital cytomegalovirus (CMV) infection Rev Med Virol 2007 17 4 253 276 10.1002/rmv.535 17579921

[B4] Liangzhen Wen Shengmei Wu Shengmin Lu The epidemiological study on human cytomegalovirus infection of pregnant women and the maternal-fetal transmission in three Chinese metropolls Chin J Obstet Gynecol 1996 31 12 714 717 9387510

[B5] Wen LZ Xing W Liu LQ AO LM Chen SH Cytomegalovirus infection in pregnancy International Journal of Gynecology and Obstetrics 2002 79 2 111 116 10.1016/S0020-7292(02)00239-4 12427394

[B6] Zhang XW Li F Yu XW Physical and intellectual development in children with asymptomatic congenital cytomegalovirus infection: a longitudinal cohort study in Qinba mountain area J Clin Virol 2007 40 3 180 5 10.1016/j.jcv.2007.08.018 17919973

[B7] Pass RF Congenital cytomegalovirus infection and hearing loss Herpes 2005 12 50 55 16209862

[B8] Fowler KB Boppana SB Congenital cytomegalovirus (CMV) infection and hearing deficit J Clin Viro 2006 35 226 231 10.1016/j.jcv.2005.09.016 16386462

[B9] Morton CC Nance WE Newborn hearing screening - a slient revolution N Engl J Med 2006 354 2151 2164 10.1056/NEJMra050700 16707752

[B10] Ogawa H Suzutani t Baba Y Etiology of severe sensorineural hearing loss in children: independent impact of congenital cytomegalovirus infection and GJB2 mutations J Infect Dis 2007 195 782 788 10.1086/511981 17299707

[B11] Numazaki K Fujikawa T Chronological changes of incidence and prognosis of children with asymptomatic congenital cytomegalovirus infection in Sapporo BMC infect Dis 2004 4 22 10.1186/1471-2334-4-22 15236662 PMC481070

[B12] Morita M Morishima T Yamazaki T Chiba S Kawana T Clinical survey of congenital cytomegalovirus infection in Japan Acta Paediatr/Jpn 1998 40 432 436 10.1111/j.1442-200x.1998.tb01963.x 9821701

[B13] Barbi M Binda S Caroppo S Ambrosetti U Corbetta C Sergi P A wider role for congenital cytomegalovirus infection in sensorineural hearing loss Pediatr, Infect Dis J 2003 22 39 42 10.1097/00006454-200301000-00012 12544407

[B14] Kaufmann P Davidoff M The guinea-pig placenta Adv Anat Embryol Cell Biol 1977 53 5 91 331890 10.1007/978-3-642-66618-6

[B15] Leiser R Kaufmann P Placental structure: in a comparative aspect Exp Clin Endocrinol 1994 102 122 134 10.1055/s-0029-1211275 7995333

[B16] Donnelly TM Brown CJ Guinea pig and chinchilla care and husbandry Vet Clin N Am Exot Anim Pract 2004 7 351 373 10.1016/j.cvex.2004.02.006 15145394

[B17] Woolf NK Koehrn FJ Congenital cytomegalovirus labyrinthitis and sensorineural hearing loss in guinea pigs J Infect Dis 1989 160 6 929 37 2555420 10.1093/infdis/160.6.929

[B18] Harutaka K Sato Y Tsutsui Y Pathogenesis of cytomegalovirus-associated labyrinthitis in a guinea pig model J Microbes and Infection 2007 9 183 191 10.1016/j.micinf.2006.11.004 17208485

[B19] Ren-Yong Li Tsutsui Y Growth retardation and microcephaly induced in mice by placental infection with murine cytomegalovirus Teratology 2000 62 79 85 10.1002/1096-9926(200008)62:2<79::AID-TERA3>3.0.CO;2-S 10931504

[B20] Chen Li Suhua Chen Haizhi Liu Juanjuan Chen Primary studies on the establishment of murine cytomegalovirus intrauterine model Chinese Journal of Birth Health & Heredity 2006 14 3 55 56 63

[B21] Keil GM Ebeling-Keil A Koszinowski UH Sequence and structural organization of murine cytomegalovirus immediate-early gene 1 J Virol 1987 61 6 1901 8 3033321 10.1128/jvi.61.6.1901-1908.1987 PMC254196

[B22] Dahle AJ Fowler KB Wright JD Longitudinal investigation of hearing disorders in children with congenital cytomegalovirus J Am Acad Audiol 2000 11 283 290 10821506

[B23] Hicks T Fowler K Richardson M Dahle A Adams L Pass R Congenital cytomegalovirus infection and neonatal auditory screening J Pediatr 1993 123 779 782 10.1016/S0022-3476(05)80859-5 8229490

[B24] Williamson WD Percy AK Yow MD Gerson P Catlin FI Koppelman ML Thurber S Asymptomatic congenital cytomegalovirus infection. Audiologic, euroradiologic, and neurodevelopmental abnormalities during the first year Am J Dis Child 1990 144 1365 1368 2173889 10.1001/archpedi.1990.02150360091031

[B25] Davis LE Johnsson LG Kornfeld M Cytomegalovirus labyrinthitis in an infant: morphological, virological, and immunofluorescent studies J Neuropathol Exp Neurol 1981 40 1 9 19 6259297

[B26] Rarey KE Davis LE Temporal bone histopathology 14 years after cytomegalic inclusion disease: a case study Laryngoscope 1993 103 8 904 9 10.1288/00005537-199308000-00012 8395628

[B27] Sugiura S Yoshikawa T Nishiyama Y Morishita Y Sato E Hattori T Nakashima T Detection of human cytomegalovirus DNA in perilymph of patients with sensorineural hearing loss using real-time PCR J Med Virol 2003 69 72 75 10.1002/jmv.10263 12436480

[B28] Tsutsui Yoshihiro Effects of cytomegalovirus infection on embryogenesis and brain development Congenital Anomalies 2000 49 47 55 10.1111/j.1741-4520.2009.00222.x 19489954

